# Overexpression of PD‐L1 in gingival basal keratinocytes reduces periodontal inflammation in a ligature‐induced periodontitis model

**DOI:** 10.1002/JPER.21-0017

**Published:** 2021-06-05

**Authors:** Keeratika Wongtim, Eri Ikeda, Tatsukuni Ohno, Shigenori Nagai, Shigeru Okuhara, Keitetsu Kure, Miyuki Azuma

**Affiliations:** ^1^ Department of Molecular Immunology Graduate School of Medical and Dental Sciences Tokyo Medical and Dental University Tokyo Japan; ^2^ Department of Periodontology Graduate School of Medical and Dental Sciences Tokyo Medical and Dental University Tokyo Japan; ^3^ Department of Molecular Craniofacial Embryology Graduate School of Medical and Dental Sciences Tokyo Medical and Dental University Tokyo Japan

**Keywords:** gingivitis, immunology, pathogenesis of periodontal disease(s), periodontitis, programmed cell death‐1 ligand 1 (pd‐l1)

## Abstract

**Background:**

The immune checkpoint programmed cell death 1 (PD‐1): PD‐1 ligand 1 (PD‐L1) pathway plays a crucial role in maintaining immune tolerance and preventing tissue damages by excessive immune responses. PD‐L1 is physiologically expressed and upregulated in keratinocytes (KCs) in the oral cavity. We here investigated the contribution of PD‐L1 that was overexpressed in gingival basal KCs in a ligature‐induced periodontitis model.

**Methods:**

Wild‐type (WT) BALB/c and K14/PD‐L1 transgenic (tg) mice, in which PD‐L1 was overexpressed in basal KCs under control of the keratin 14 promoter, were used. To induce periodontitis, a 9‐0 silk ligature was placed around the upper right second molar, and lipopolysaccharide from *Porphyromonas gingivalis* was applied on the suture. Gingival tissues were collected on day 7, after which histological analyses were performed, including by hematoxylin and eosin and tartrate‐resistant acid phosphate staining (TRAP) and quantitative PCR for proinflammatory cytokines and bone metabolism‐related genes. Alveolar bone loss at 7 weeks after ligature placement was assessed by micro‐computed tomography analysis.

**Results:**

PD‐L1 was overexpressed in the basal KCs of all gingival epithelia in K14/PD‐L1tg mice. Early ligature‐induced periodontal inflammation, as assessed based on histological changes, elevation of proinflammatory cytokine (IL‐1β, IL‐6, TNF‐α) expression, periodontal ligament degeneration, and osteoclastogenesis as assessed by Rankl and Opg expression and TRAP+ cells, was markedly impaired in K14/PD‐L1tg mice. Alveolar bone resorption at a late time point was also clearly minimized in K14/PD‐L1tg mice.

**Conclusion:**

Overexpression of PD‐L1 in gingival basal keratinocytes in K14/PD‐L1tg mice reduces periodontal inflammation and alveolar bone resorption in a ligature‐induced periodontitis model.

## 1 INTRODUCTION

Multiple T cell cosignal pathways control the balance of immunity and tolerance^1^. In particular, the immune checkpoint programmed cell death 1 (PD‐1)/PD‐1 ligand 1 (PD‐L1) pathway plays a crucial role in preventing tissue damages because of excessive immune responses at the inflammatory tissues.^2^, ^3^ PD‐L1 (also known as B7‐H1) is often induced on non‐lymphoid tissues cells and tumor cells by inflammatory cytokines such as IFN‐γ, TNF‐α, and IL‐1, as well as toll‐like receptor‐mediated signaling.^3^ Keratinocytes (KCs) are the lining cells of stratified squamous epithelium in the skin and the oral mucosae. Our group and others showed PD‐L1 expression in oral KCs and periodontal ligament (PDL) cells in healthy controls and those with diseases.^4^, ^5^, ^6^, ^7^ We previously generated transgenic mice (tg) that overexpressed PD‐L1 in basal KCs under the control of the keratin 14 (K14) promoter (K14/PD‐L1tg) and demonstrated that basal KC‐associated PD‐L1 inhibited skin contact hypersensitivity via direct interactions with PD‐1^+^ CD8^+^ T cells.^8^ PD‐L1 in the gingival epithelium and the dorsal tongue epithelium is up‐regulated by environmental physiological stimuli and inhibits local inflammation via interactions with tissue recruiting PD‐1^+^ CD4^+^ T cells.^6^

Periodontal disease is a common oral disease associated with the interactions between dysbiotic oral microbiota and host immune responses.^9^ Periodontitis is initiated by dysbiotic bacterial communities forming on subgingival tooth surfaces and undergoes a transition to long‐lasting chronic inflammation with alveolar bone loss. Ligature‐induced periodontitis mice models have been more frequently used to study periodontal disease mechanisms and to test the potential of novel therapeutic methods.^10^, ^11^ Although involvement of local PD‐L1 in the progression of periodontitis has been suggested,^5^, ^12^ the functional contribution of PD‐L1 induced on gingival KCs to periodontal inflammation has not been investigated, because PD‐L1 is up‐regulated in various non‐immune tissue cells as well as immune cells under the inflammatory conditions. In this study, to investigate the role of overexpressing PD‐L1 in gingival basal KCs, we compared periodontal inflammatory responses at an earlier time point (7 days) and alveolar bone resorption at a late time point (7 weeks) between wild‐type (WT) and K14‐PD‐L1tg mice in a ligature‐induced periodontitis model.

## 2 MATERIALS AND METHODS

### 2.1 Mice

K14/PD‐L1tg mice (*n* = 40) with a BALB/c background were generated and bred, as previously described,^8^ and WT BALB/c mice (*n* = 40) were purchased from a supplier of laboratory animals*. All mice were maintained under specific pathogen‐free conditions at Tokyo Medical and Dental University (Tokyo, Japan), and female 7‐ to 8‐week‐old mice were used. All experimental procedures were reviewed and approved by the animal care and use committee of Tokyo Medical and Dental University (A2019206, A20180262).

### 2.2 Immunohistochemistry

For detection of PD‐L1, cryostat sections of gingival tissues were subjected to enzymatic immunohistochemistry, as previously described.^8^ The sections were first incubated with anti‐PD‐L1 (MIH6) monoclonal antibody or control rat IgG and subsequently with a biotinylated anti‐rat IgG†. Antibody binding was detected using an avidin‐biotin‐peroxidase complex system‡, visualized with the substrate diaminobenzidine, and counterstained with hematoxylin.

### 2.3 Ligature‐induced periodontitis

To evaluate early and late time points of immune‐related periodontal changes, we utilized a 9‐0 silk ligature model.^13^, ^14^ Although the experimental period in most ligature‐induced periodontitis model using 5‐0 or 6‐0 silk thread are around 7 to 14 days,^10^, ^11^, ^15^ we prefer to keep enough experimental period to discriminate acute inflammation and chronic progression phases and to reduce a possibility that a large ligature placement around the tiny mouse molar induces an artificial rapid mechanical injury with radical bone resorption. Under the anesthesia with ketamine and xylazine, a 9‐0 silk suture was placed in the gingival sulcus around the maxillary right second molar (M2) and tied on the palatal side using a surgeon's knot under a stereomicroscope, as previously described.^13^, ^14^ After suture placement, lipopolysaccharide (LPS) from *Porphyromonas gingivalis* 381 strain^16^ (1 μg diluted with 5 μL phosphate‐buffered saline) was topically applied on the sutures for 4 consecutive days. The contralateral left M2 served as the unligated /non‐treated control. Ligature status was observed every other day. To assess acute and chronic events of periodontal inflammation, mice were sacrificed by cervical dislocation at 7 days and 7 weeks after ligature placement, and periodontal tissues and maxilla samples were harvested.

### 2.4 Histological analysis

**FIGURE 1:**
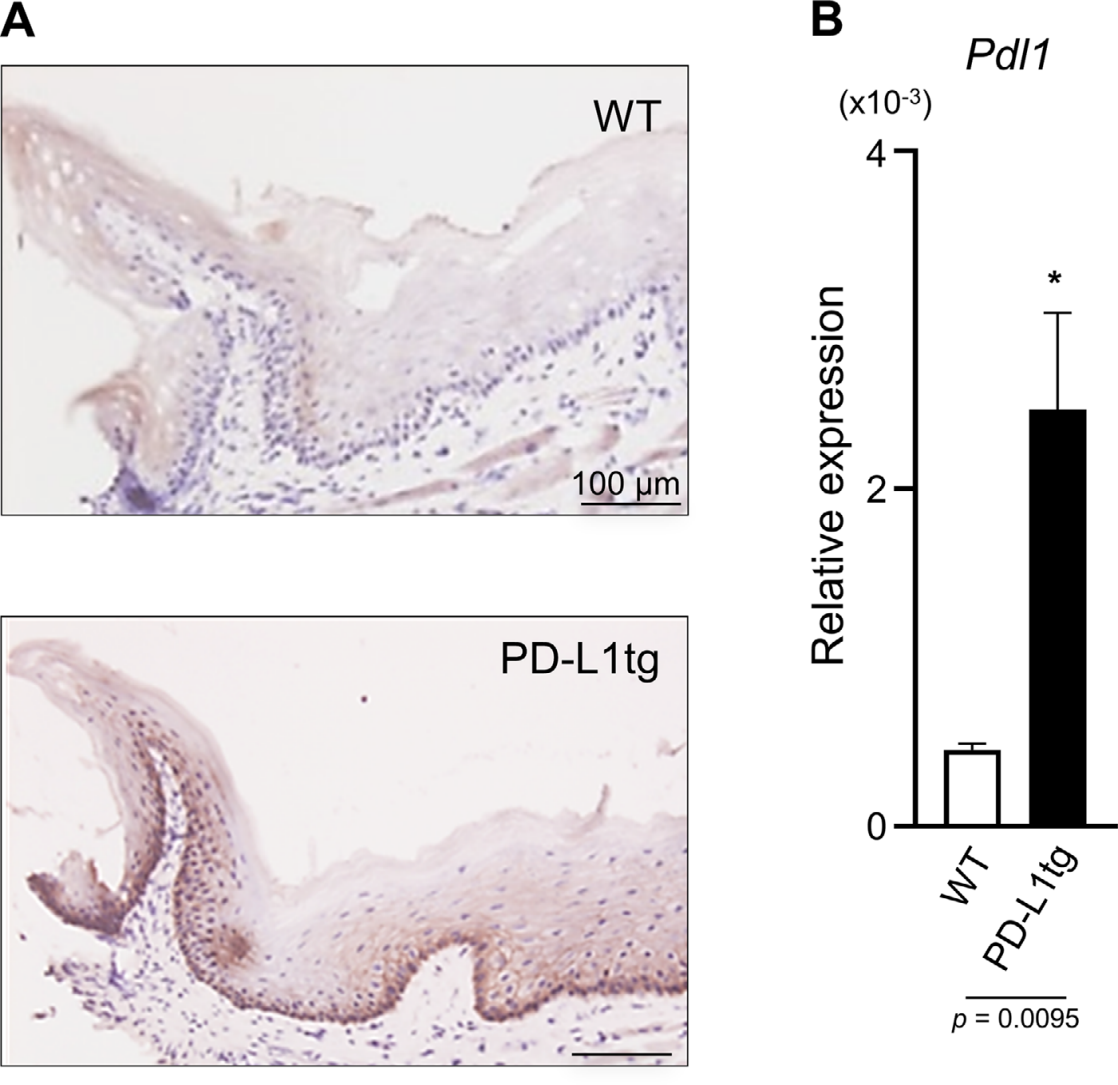
Overexpression of PD‐L1 in the basal layer of the gingival epithelium in K14/PD‐L1tg mice. (**A**) Fresh frozen gingival tissue sections from WT and K14/PD‐L1tg mice were stained with anti‐PD‐L1 monoclonal antibody. Scale bar = 100 μm. (**B**) Gingival tissues from WT and K14/PD‐L1tg mice were subjected to qPCR for pdl1(cd274) and Gapdh. Relative expression against Gapdh is shown. Values are presented as the mean ± standard deviation (SD) from 4‐6 mice. *Statistically different compared to WT mice (Mann‐Whitney U test; P < 0.05)

**FIGURE 2:**
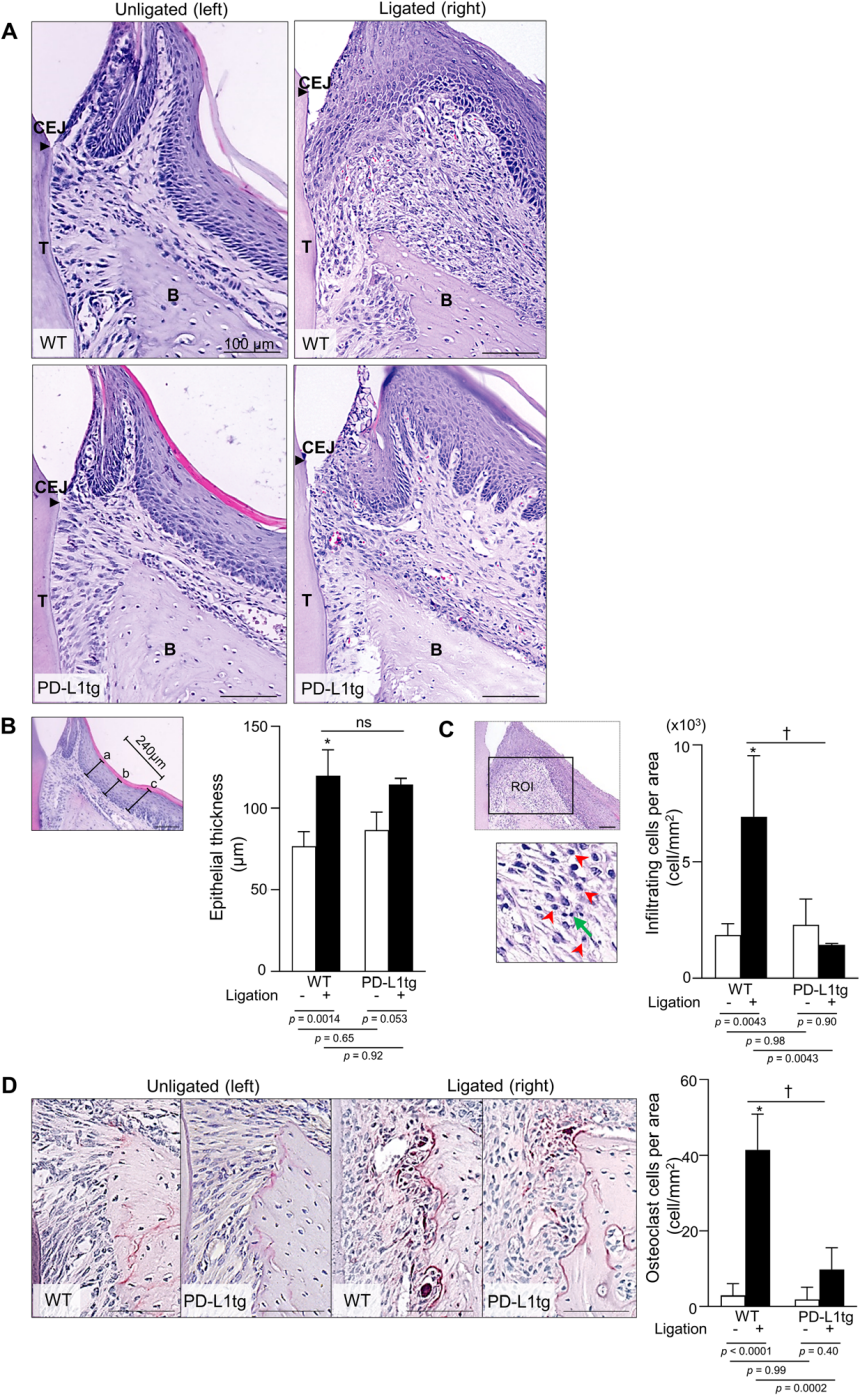
Ligature‐induced histological changes are impaired in K14/PD‐L1tg mice. (**A, D**) Formalin‐fixed paraffin‐embedded sections of samples collected on day 7 were subjected to H&E (A) and TRAP (D) staining. Representative images of unligated and ligated M2s from WT and K14/PD‐L1tg mice from each group of 4 mice are shown. Similar results were obtained from independent two experiments. B, alveolar bone; CEJ, cement‐enamel junction (arrow heads); T, tooth. Scale bars = 100 μm. (**B, C, D**) Semiquantitative histological assessment of the epithelial thickness of the palatal mucosa (B) and measurement of the number of infiltrating cells in subepithelial connective tissues (C) and the number of osteoclasts in tooth side of alveolar bone (D). (B) The mean epithelial thickness from the indicated three points (left panel) was obtained. Values presented are the mean ± SD from 3‐4 mice. (C) How to place the ROI is shown in the 200x magnification image (upper panel) and representative neutrophils (red arrowhead) and lymphocytes (green arrow) are shown (lower panel). Values are the mean ± SD from 3‐4 mice. (D) Representative TRAP staining from each group is shown in the left. The number of osteoclasts in the PDL was counted. Values presented as the mean ± SD from 3‐4 mice. *Statistically different compared to WT mice (P < 0.05). †Statistically difference on the ligated side between WT and K14/PD‐L1tg mice (two‐way ANOVA and Tukey test; P < 0.05). ns; no statistically difference

Hemi‐maxilla samples were fixed in 10% neutral buffered formalin, decalcified in 10% ethylenediaminetetraacetic acid (pH 7.4) for 3 weeks, and embedded in paraffin. Microtome‐cut 6‐μm sections in the mesial‐distal plane were stained with hematoxylin and eosin (H&E). For identification of bone‐resorbing osteoclasts, tartrate‐resistant acid phosphate (TRAP) staining was performed as previously described.^17^ Briefly, sections were stained using 1.3‐mM naphthol AS‐MX phosphate§, 1.6‐mM Fast Red Violet LB salt^§^, and 50‐mM sodium tartrate in 100‐mM acetic buffer (pH 5.2), and counterstained with hematoxylin. Representative stained slide from individual mouse was selected and all images were acquired using a quantitative pathology workstation**. Semi‐quantitative evaluation of the epithelial thickness in the attached gingiva; the numbers of inflammatory cells including neutrophils and lymphocytes in the subepithelial connective tissue area; and the numbers of osteoclasts in the tooth side area of alveolar bone were determined. For measurement of epithelial thickness, thickness at three points in a 240‐μm wide section of gingival epithelium as shown in the left panel of Figure 2B was assessed. In the case of wavy rete ridges, the average of the longer and shorter thickness measurements was obtained. Then, the mean value of three measurements was used as an individual datapoint. For measurement of infiltrating cells, we placed a region of interest (ROI) to include most of subepithelial connective tissues surrounded by the tooth, alveolar bone crest and gingival epithelium as shown in the left panel of Figure 1C, and then based on morphological shape and H&E staining pattern, we counted mononuclear lymphocytes and neutrophils as infiltrating cells at the whole image of 400× magnification. The area of subepithelial connective tissue space was measured by a quantitative pathology workstation^**^ (1 pixel is equivalent to 0.0625 μm^2^). The final values for infiltrating cells per area were obtained by total infiltrating cell number divided by the area (cells/mm^2^).

For semiquantitative evaluation of osteoclast cells, TRAP‐positive cells with three or more nuclei were considered osteoclasts. We counted TRAP^+^ osteoclasts in the PDL at the 200× magnification. The final values for osteoclast cells per area were obtained by total osteoclast cell number divided by the area (cells/mm^2^).

### 2.5 Quantitative PCR

Gingival tissues around the maxillary M2 were obtained after 7 days. Total RNA was extracted, and quantitative real‐time PCR (qPCR) analysis for *pdl1 (cd274)*, *IL‐1β*, *IL‐6*, *TNF‐α*, receptor activator of nuclear factor kappa‐β ligand (*Rankl*), and *osteoprotegerin* (*Opg*) was performed as previously described.^18^ The following primer sets were used: *glyceraldehyde 3‐phosphate dehydrogenase* (*Gapdh*), forward, *5′‐*GCATGGCCTTCCGTGTTCCT‐3′ and reverse, *5′‐*GGTCCTCAGTGTAGCCCAAGATGC‐3′; *pdl1, forward, 5′‐*TGCGGACTACAAGCGAATCA‐3′ and reverse, 5′‐GATCCACGGAAATTCTCTGGTT

‐3′; Il1b, forward, 5′‐GTGACGTTCCCATTAGACAGC‐3′ and reverse, *5′‐*CCCAAGGCCACAGGTATTT‐3′; Il6, forward, 5′‐GAGATACAAAGAAATGATGGATGC‐3′ and reverse, 5′‐ACTCTGGCTTTGTCTTTCTTGTTA‐3′; Tnfa, forward, 5′‐CATCAAGGACTCAAATGGG‐3′ and reverse, 5′‐TGGAAAGGTCTGAAGGTAGG‐3′, *Rankl*, forward, 5′‐AGCCATTTGCACACCTCAC‐3′ and reverse, 5′‐CGTGGTACCAAGAGGACAGAGT‐3′; *Opg*; forward, 5′‐ACCCAGAAACTGGTCATCAGC ‐3′ and reverse, 5′‐CTGCAATACACACACTCATCACT‐3′. Data are presented as ratios relative to *Gapdh*.

### 2.6 Alveolar bone assessment using micro‐computed tomography

Hemi‐maxilla samples resected at 7 weeks were defleshed and immersed in 1% proteinase K buffer with 30% hydrogen peroxide. Dried skulls were stained with methylene blue, and the samples were scanned in all three spatial planes at a resolution of 1024 × 1024 × 1024 voxels using micro‐computed tomography (micro‐CT)††and 3D bone morphometry‡‡, as described previously.^19^ To evaluate alveolar bone resorption, the distance between the cement‐enamel junction (CEJ) and the alveolar bone crest (ABC) on the palatal side of M2 were measured at three sites corresponding to the mesio‐palatal (MP) cusp, palatal groove (P) and disto‐palatal (DP) cusp for each sample. CEJ‐ABC distances measured at the three sites on the ligated side were subtracted from the respective CEJ‐ABC distances measured on the contralateral unligated side in the same mouse.

### 2.7 Statistical analysis

Statistical analysis was performed using the Mann‐Whitney *U* test or two‐way analysis of variance (ANOVA) and Tukey test with a statistical software§§. Values of *P* < 0.05 were considered to indicate statistical significance.

## 3 RESULTS

### 3.1 PD‐L1 is overexpressed in basal cells of the gingival epithelium in K14/PD‐L1tg mice

First, we compared the protein and mRNA expression levels of PD‐L1 in gingival tissues from WT and K14/PD‐L1tg mice using enzymatic immunohistochemistry and qPCR, respectively. In the gingival tissues from WT mice, a weak PD‐L1 expression was observed in the prickly layer of gingival epithelium and in immune cells of the lamina propria (Figure 1A). In the gingival epithelium from K14/PD‐L1tg mice, we observed strong PD‐L1 expression in the basal layer and moderate expression in the prickly layer. The qPCR analyses showed that the mRNA expression level of PD‐L1 in the gingival tissues from K14/PD‐L1tg mice was ≈5‐fold higher than that from WT mice (Figure 1B). Thus, we confirmed the overexpression of PD‐L1 in basal cells of the gingival epithelium in K14/PD‐L1tg mice.

### 3.2 Ligature‐induced histopathological changes are limited in K14/PD‐L1tg mice

To examine the effects of overexpressing PD‐L1 in the gingival basal cells, we compared periodontal inflammation between WT and K14/PD‐L1tg mice in a 9‐0 ligature‐induced periodontitis model. Histopathological changes in the periodontal tissues were analyzed on day 7. At the unligated side, there were no remarkable differences in the junctional epithelium, subepithelial connective tissues, and alveolar bone between WT and K14/PD‐L1tg mice (Figure 2A). The epithelial attachment at the CEJ on the unligated side was kept intact in both groups. Ligation induced remarkable histological changes in both WT and K14/PD‐L1tg mice on day 7. On the ligated side in WT mice, the junctional epithelium was detached, and degeneration of junctional epithelium occurred. In addition, abundant vasculature, degeneration of connective tissue cells, and enlargement of intercellular spaces were observed. Of note, these changes were clearly less on the ligated side of K14/PD‐L1tg mice. Ligature/LPS treatment in WT mice increased the number of epithelial layers and disarrangement of basal KCs, and disrupted the construction of sulcular epithelium and free gingiva. Elongation of rete pegs with well‐organized basal cells was seen in some of K14/PD‐L1tg mice. Semiquantitative analyses of epithelial thickness in the attached gingiva showed that the ligature treatment significantly increased the mean thickness in WT mice, but no clear differences were observed between WT and K14/PD‐L1tg mice on the ligated side (Figure 2B). In the subepithelial connective tissues, abundant neutrophil and moderate lymphocyte infiltration was seen on the ligated side of WT mice. Semiquantitative analyses based on morphological assessment revealed that the number of infiltrating cells, including neutrophils and lymphocytes, was markedly increased on the ligated side of WT mice, but this was significantly impaired in K14/PD‐L1tg mice (Figure 2C). This suggests that the recruitment of infiltrating cells was reduced in K14/PD‐L1tg mice.

Next, we examined the region of the PDL and alveolar bone. The alignment of PDL cells on the unligated side in both groups was almost intact, but the ligation induced degeneration of PDL with severe disarrangement of PDL cells, especially near the alveolar crest in WT mice (Figure 2A). Although the distance from the CEJ to the alveolar bone crest was not obviously changed, we observed clear alveolar bone defects with the appearance of TRAP^+^ multinuclear cells at the PDL in the ligated molar from WT mice (Figure 2A,D). These ligature‐induced changes were markedly limited in K14/PD‐L1tg mice (Figure 2D). These results indicate that the ligature‐induced osteoclast formation observed in WT mice was markedly impaired in K14/PD‐L1 mice. Taken together, our histopathological results demonstrate that the ligature‐induced inflammatory changes in the gingival epithelium, subepithelial connective tissues, PDL, and alveolar bone observed in WT mice were clearly reduced in K14/PD‐L1tg mice.

### 3.3 Proinflammatory cytokines and the RANKL/OPG ratio are reduced in the ligated side of gingival tissues from K14/PD‐L1tg

**FIGURE 3:**
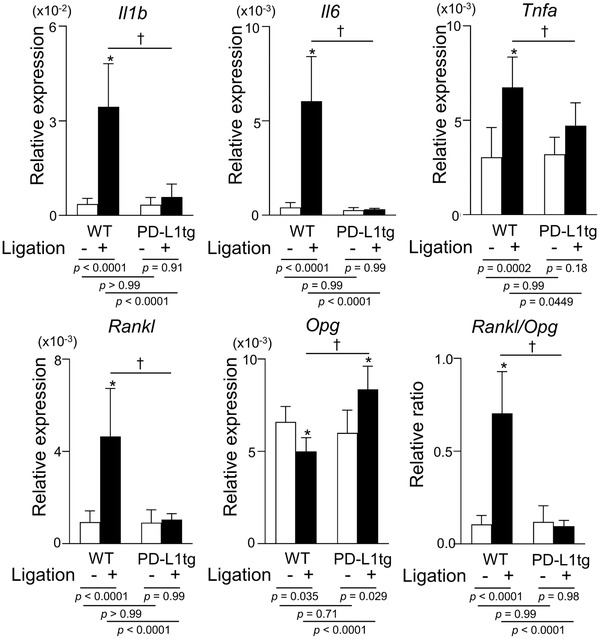
Impaired expression of proinflammatory cytokines and the *Rankl/Opg* ratio in ligated gingival tissues from K14/PD‐L1tg. Gingival tissues around M2s at 7 days after ligation were subjected to qPCR. Values are presented as the mean ± SD (*n* = 6‐7) from two independent experiments. Relative expression against *Gapdh* is shown. *Significantly different compared to WT mice (*P* < 0.05). ^†^Significant difference on the ligated side between WT and K14/PD‐L1tg mice (two‐way ANOVA and Tukey test; *P* < 0.05)

To further confirm the differences between WT and K14/PD‐L1tg mice, we evaluated the mRNA levels of proinflammatory cytokines (IL‐1β, IL‐6, and TNF‐α) and bone metabolism‐related genes (*Rankl* and *Opg*) in the gingival tissues surrounding M2 on day 7. We observed low levels of *IL‐1β, IL‐6, and TNF‐α* mRNA in the unligated gingival tissues from both WT and K14/PD‐L1tg mice (Figure 3). The ligature treatment markedly increased their expression in WT mice, but it did not induce a clear elevation of the three cytokines in K14/PD‐L1tg mice. Instead, their expression levels in K14/PD‐L1tg mice were significantly lower, indicating reduced inflammatory cytokine expression. RANKL is a critical factor for osteoclast formation and OPG is a decoy receptor for RANKL that inhibits osteoclast differentiation and function.^20^, ^21^ Although we observed a minimal level of *Rankl* expression in unligated gingival tissues from both groups, ligature treatment in WT but not in K14/PD‐L1tg mice, markedly increased its expression. By contrast, *Opg* expression was reversely increased in ligated gingival tissues from K14/PD‐L1tg mice. The ratio of *Rankl/Opg* expression was significantly lower in ligated gingival tissues from K14/PD‐L1tg mice, suggesting impaired generation of osteoclasts. (Figure 3)

### 3.4 Alveolar bone is well‐preserved on the ligated side of M2 in K14/PD‐L1tg mice

**FIGURE 4:**
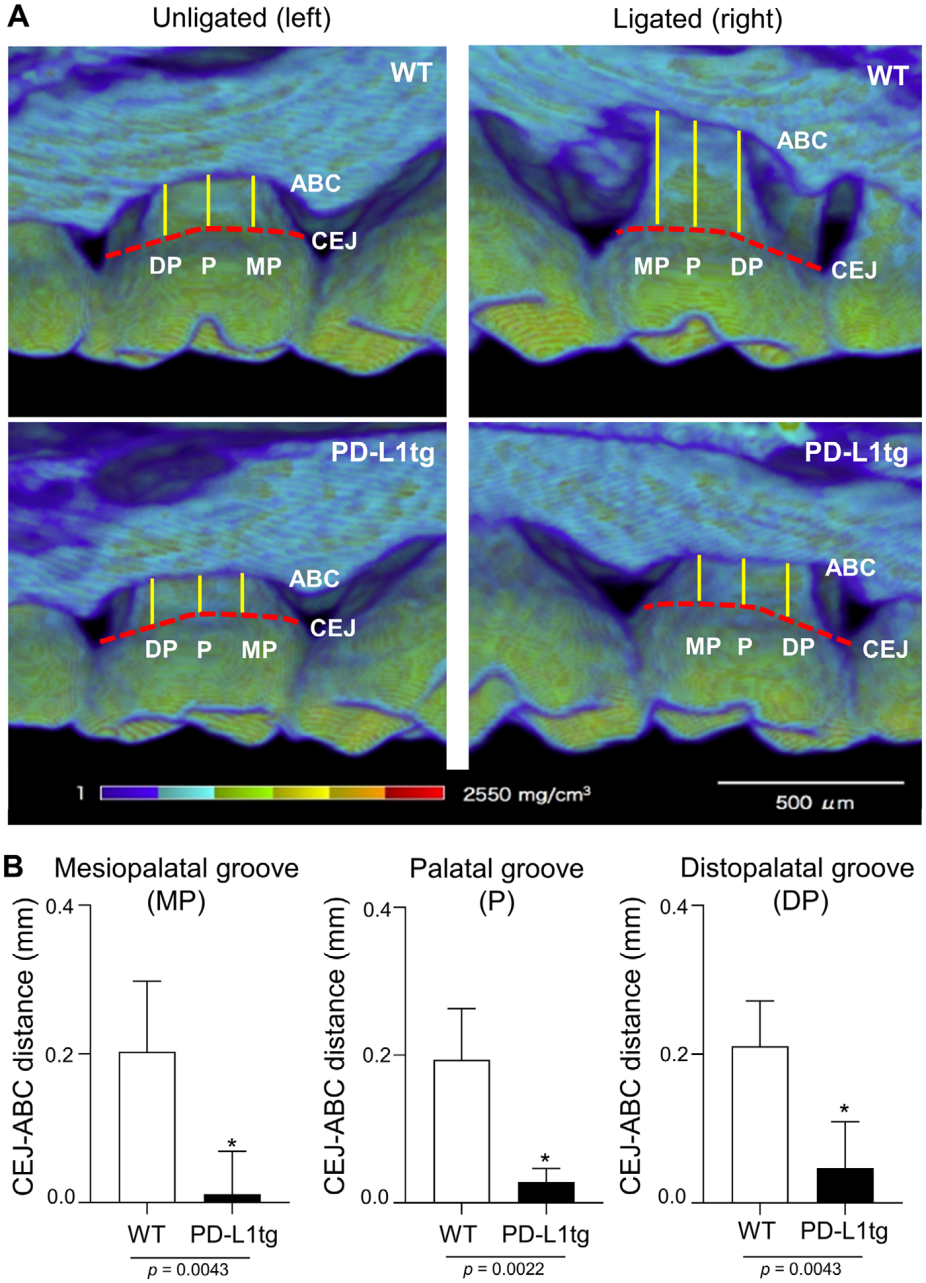
Alveolar bone in the ligated M2 is well‐preserved in K14/PD‐L1tg mice. (**A**) Representative micro‐CT images of the palatal view of unligated and ligated M2s at 7 weeks after ligature placement. ABC, alveolar bone crest; CEJ, cement‐enamel junction; DP, disto‐palatal cusp; MP, mesio‐palatal cusp; P, palatal groove. Scale bar = 500 μm. (**B**) The relative CEJ‐ABC distance at the MP, P, and DP sites are shown. Values are presented as the mean ± SD from each group of six mice analyzed in two independent experiments. *P < 0.05 compared to WT mice (Mann‐Whitney *U* test)

Alveolar bone loss is a hallmark of periodontitis progression. We assessed alveolar bone loss at the palatal side of M2 using micro‐CT at 7 weeks after ligature placement. Among four representative images, obvious bone loss can be seen in the image of a ligated M2 from a WT mouse compared to the other three images (Figure 4A). Analyses of the distance between the CEJ and the ABC measured at three sites (MP, P, DP) revealed that the mean relative CEJ‐ABC distances were consistently smaller in K14/PD‐L1tg mice (Figure 4B), indicating well‐preserved alveolar bone despite the ligature placement. These results indicate that the progression to periodontitis with alveolar bone loss was prevented in K14‐PD‐L1tg mice.

## 4 DISCUSSION

We demonstrated that ligature‐induced early periodontal inflammation (day 7), as assessed based on histological changes, expression of proinflammatory cytokines and bone metabolism‐related genes, PDL degeneration, and osteoclast formation, was reduced in K14/PD‐L1tg mice. Progression to periodontitis as assessed by alveolar bone resorption at the late time point (7 weeks) was also markedly minimized.

Despite overexpression of PD‐L1 in the basal KCs of sulcular epithelium as well as free and attached gingiva on the unligated gingival side, no clear differences in epithelial cell status and infiltrating cells in the subepithelial connective tissues were observed between WT and K14/PD‐L1tg mice. This indicates that the local PD‐1/PD‐L1 pathway is not involved in the physiological steady state of the gingiva, although the gingival surface is consistently exposed to rich and diverse commensal microbes.

Once inflammation occurred because of the ligature/LPS treatment, the differences between WT and K14/PD‐L1tg mice became obvious, even at the early point (day 7). Ligature placement induces a rapid increase in the expression of proinflammatory cytokines such as IL‐6, IL‐1β, and TNF‐α within 24 hours.^15^ Initially secreted proinflammatory cytokines quickly stimulate the activation of surrounding stromal cells such as KCs and resident immune cells, and the secreted chemokines and cytokines from these cells further recruit additional inflammatory cells. We consistently observed up‐regulation of infiltrating immune cells, expression of proinflammatory cytokines (IL‐1β, IL‐6, and TNF‐α), and osteoclastogenesis in the ligated periodontal tissues, and these observations were much rarer in the gingival tissues overexpressing PD‐L1 in basal KCs. One question then is which cells can express a counter‐receptor of PD‐1 and interact with PD‐L1 on basal KCs. In the ligated periodontal tissues, we found an increase in infiltrating cells including neutrophils, macrophages, and lymphocytes. These three populations of cells may be possible candidates that can interact with PD‐L1‐expressing basal KCs. Although PD‐1 expression on T cells and their functions are well understood, PD‐1 is also inducible on macrophages and neutrophils under certain pathological conditions such as the microbial response and in the tumor microenvironment.^22^, ^23^, ^24^ PD‐1 then modulates their phagocytosis capabilities and the induction of innate inflammatory responses. It is possible that the binding of PD‐L1 on proliferating basal KCs to PD‐1 on macrophage/neutrophils may convert their inflammatory property to that of tolerogenicity.

Another reasonable explanation is that PD‐1‐expressing gingival T cells may interact with PD‐L1 on KCs, which render their effector function non‐functional exhausted T cells. Human healthy gingival tissues contain a predominance of T cells in CD45^+^ hematopoietic‐origin cells,^25^ including one third of Foxp3^+^ regulatory T cells, which comprise one‐third of all cells, followed by CD8^+^ T cells. Most gingival T cells exhibit the CD69^+^ resident memory phenotype.^25^ and this phenotype increases in periodontitis tissues.^26^, ^27^ At the frontline of mucosal barriers in the lung and gut, CD8^+^ tissue‐resident memory T (Trm) cells play an important role in cognate interactions with local epithelial cells in the context of major histocompatibility complex (MHC) class I signaling.^28^ PD‐1 on CD8^+^ Trm cells is quickly induced by T‐cell receptor (TCR)/MHC‐mediated signals together with surrounding inflammatory stimuli. PD‐1 expression is often most prominent on cells with Trm cell characteristics, and the blockade of PD‐1 reinforces the conversion of exhausted Trm cells to functional effector T cells in the tumor microenvironment.^29^ Presently, there is only one report showing PD‐1 expression in inflamed gingival CD4^+^ and CD8^+^ T cells based on immunofluorescence confocal microscopy.^30^ Further studies are required to demonstrate the existence of PD‐1^+^ Trm cells in gingival tissues and their functional involvement in the regulation of periodontal inflammation. Local inactivation of CD8^+^ T cells by PD‐L1‐expressing KCs may further downregulate inflammatory responses mediated by CD4^+^ T cells and macrophages, resulting in the prevention of RANKL‐dependent osteoclastogenesis.^20^, ^21^ It should be noted that our observation of limited osteoclastogenesis in K14/PD‐L1tg mice even on day 7 indicates that local PD‐1/PD‐L1‐mediated regulation is an upstream event during the process of gingival inflammation.

## 5 CONCLUSIONS

In summary, our results demonstrated protective effects in the early phase of gingival inflammation and periodontal destruction in K14/PD‐L1tg mice in which PD‐L1 was overexpressed in the basal KCs of the gingival epithelium. These data suggest that overexpressing PD‐L1 in basal KCs plays an important role in the regulation of gingival inflammation and the progression to periodontitis.

## CONFLICT OF INTEREST

All authors declare no conflict of interest.

## AUTHOR CONTRIBUTIONS

W.K. and M.A. contributed to conception and design of the research, interpretation and wrote the paper. W.K., T.O., E.I., S.O., and K.K. performed the experiments, data acquisition and analysis. S.N. contributed to the critical discussion of the research and manuscript preparation.

## Footnotes

* Japan SLC, Hamamatsu, Japan.

† Vector Laboratories, Burlingame, CA.

‡ VECSTATIN; Vector Laboratories.

§ Sigma‐Aldrich; St Louis, MO.

** Mantra; Akoya Biosciences; Marlborough, MA.

†† InspeXio SMX‐100CT; Shimadzu, Tokyo, Japan.

‡‡ TRI/3D‐BON software; RATOC System Engineering; Tokyo, Japan.

§§ Prism 8 software; GraphPad Software Inc.; La Jolla, CA.
